# Central Role of Oxidative Stress in Age-Related Macular Degeneration: Evidence from a Review of the Molecular Mechanisms and Animal Models

**DOI:** 10.1155/2020/7901270

**Published:** 2020-02-10

**Authors:** Samuel Abokyi, Chi-Ho To, Tim T. Lam, Dennis Y. Tse

**Affiliations:** ^1^School of Optometry, The Hong Kong Polytechnic University, Hong Kong; ^2^Department of Optometry, University of Cape Coast, Ghana

## Abstract

Age-related macular degeneration (AMD) is a common cause of visual impairment in the elderly. There are very limited therapeutic options for AMD with the predominant therapies targeting vascular endothelial growth factor (VEGF) in the retina of patients afflicted with wet AMD. Hence, it is important to remind readers, especially those interested in AMD, about current studies that may help to develop novel therapies for other stages of AMD. This study, therefore, provides a comprehensive review of studies on human specimens as well as rodent models of the disease, to identify and analyze the molecular mechanisms behind AMD development and progression. The evaluation of this information highlights the central role that oxidative damage in the retina plays in contributing to major pathways, including inflammation and angiogenesis, found in the AMD phenotype. Following on the debate of oxidative stress as the earliest injury in the AMD pathogenesis, we demonstrated how the targeting of oxidative stress-associated pathways, such as autophagy and nuclear factor erythroid 2-related factor 2 (Nrf2) signaling, might be the futuristic direction to explore in the search of an effective treatment for AMD, as the dysregulation of these mechanisms is crucial to oxidative injury in the retina. In addition, animal models of AMD have been discussed in great detail, with their strengths and pitfalls included, to assist inform in the selection of suitable models for investigating any of the molecular mechanisms.

## 1. Introduction

Age-related macular degeneration (AMD) is a neurodegenerative disease that affects the central retina of an aging eye, resulting in a progressive loss of vision and a common cause of visual impairment and disability in the aging population [1]. The global burden of AMD is estimated at 8.7% and dry AMD accounts for approximately 90% of the total number of people with this vision-threatening condition [2]. Currently, anti-vascular endothelial growth factor (anti-VEGF) therapy is approved only for the treatment of the wet form of AMD and involves the inhibition of VEGF from binding to VEGF receptors in the retina. The search for an effective treatment for dry AMD is still ongoing and depends on the understanding of the sequence of molecular mechanisms that are involved in the pathogenesis of this eye disease.

Studies in human populations and donor eyes from AMD patients have provided significant insight into the understanding of the pathogenesis of AMD. Evidence indicates that AMD is a multifactorial disease, having both genetic and environmental risk factors [3]. The risk of AMD is greater in persons with a family history of the disease than those without [4]. Observational studies have identified major environmental risk factors such as cigarette smoking, obesity, nutritional factors, and alcoholism [3]. However, investigation of the pathogenesis of AMD is limited by the inability to study the molecular mechanisms involved as they might have happened long before the diagnosis of the condition. Also, it is challenging to study this condition because of the complex nature of AMD which may arise from interactions among those risk factors involved. Hence, the use of animal models of retinal degeneration under controlled conditions in studying AMD provides crucial insight into the disease. In addition, the inducement of retinal degeneration in animals takes a relatively shorter time and provides prompt information than studying AMD in humans. As a result, studies on animal models have played a pivotal role in the preclinical evaluations of interventions, such as anti-VEGF treatments in neovascular AMD, before trials P of such treatments in human [5].

Experimental models of AMD have been established in many species including drosophila, mice, rats, guinea pigs, and monkeys. While the primate models may be preferable due to their similarities in retina structure and drusen formation and composition with humans [6], the longer time required for inducement and challenge in breeding them make the murine models much preferred for studying AMD because of lower cost, faster disease progression, and ease of genetic engineering. However, no existing animal model yet fully recapitulates the retinal changes found in human AMD. Notwithstanding, the rodent (murine) models show retinal changes including subretinal deposits, thickening of the Bruch's membrane (BrM), loss of retinal pigment epithelium (RPE) and photoreceptors, and choroidal neovascularization (CNV), which are the characteristics of AMD [7].

The objective of this review was to evaluate evidence in support of the involvement of oxidative stress, inflammation, dysregulated lipid metabolism, and dysregulated angiogenesis in the pathogenesis of AMD, relying on the information from human studies and existing animal models of AMD, to help illustrate the roles of these mechanisms. The strengths and pitfalls of each animal model were reviewed to assist inform in the selection of suitable models for investigating any of the molecular mechanisms. We demonstrated the primary role that oxidative stress may play in triggering each of the mechanisms and illustrated why the targeting of mechanisms including autophagy, Nrf2, and lipid metabolism in the retina might be the futuristic research direction to explore in the search of treatment for a AMD.

### 1.1. Overview of AMD Pathobiology

The histopathology of the AMD retina reveals that this ocular disease is characterized by localized destruction of the retinal layers at the macular region. Retinal changes observed in the AMD eyes are varied and include (1) loss of the RPE and photoreceptor layer, (2) accumulation of lipids and protein deposits beneath the RPE or in the BrM, and (3) choriocapillaris dropout, CNV, and disciform scarring [8, 9]. In addition to these is inflammation response through the recruitment of macrophages and microglia, and complement activation [10, 11].

Although controversial, most studies have proposed the RPE as the primary site of injury in AMD [8, 12]. The RPE *in situ* performs several functions to maintain retinal homeostasis, some of which include (1) regulating the transport of nutrients and metabolites, (2) absorption of light, (3) recycling of the retinal visual pigment for the continuity of phototransduction, and (4) phagocytosis of shed photoreceptor outer segments. Experimental data support that a malfunction of the RPE leads to retinal degeneration in animal models [13]. One of the early molecular event believed to be associated with RPE malfunction in AMD is the age-related accumulation of lipofuscin, a remnant from poorly degraded phagocytosed photoreceptor outer segments [14, 15]. Lipofuscin in the RPE may contribute to oxidative damage through the generation of free radicals, as well as inhibition of phagocytotic degradation of damaged biomolecules and organelles [16–19].

Adjacent to the RPE pathological changes in AMD eyes are extracellular deposits, which include the basal lamina deposit, basal linear deposits, and drusen. Drusen and other basal deposits in the BrM are important risk factors in the development of AMD [20]. Generally, two processes are believed to contribute in the formation of the subretinal lipid/protein deposits: (1) inefficient RPE metabolism, inefficient degradation of substrate, and/or damaged RPE cells give rise to debris, and (2) local chronic inflammation due to complement activation as activated microglia are recruited to the site of debris [6, 11, 21, 22]. Apart from the immunogenic properties, these lipid deposits become easily oxidized contributing to oxidative stress [23, 24]. In addition, the BrM serves as a semipermeable membrane facilitating the diffusion of nutrients and metabolites between the outer retina and the choriocapillaris; hence, accumulation of deposits within the BrM impairs the transportation of molecules and lead to damage of the RPE and photoreceptor layers [24, 25]. While pathological changes in the RPE may be widely accepted to be the earliest damage in AMD, it was argued by some researchers that the pathophysiology in AMD may differ between the dry AMD and wet AMD [9, 26]. Mcleod and coworkers reported that in dry AMD, regions of complete RPE atrophy in the AMD retina preceded the adjacent areas of loss in the choriocapillaris, indicating the RPE as the primary site of insult. In contrast, they observed that in wet AMD or choroidal neovascularization (CNV), choriocapillaris loss preceded RPE atrophy, implicating the choriocapillaris as the focus of injury in wet AMD, which in turn could induce hypoxia in the adjacent RPE, upregulating vascular endothelial growth factor (VEGF), and promoting CNV [9].

## 2. Molecular Mechanisms and Models of AMD

Evidence suggests that AMD is a complex disease having multiple risk factors and molecular mechanisms. The studies on experimental models of AMD suggest that these molecular mechanisms involved in AMD could be categorized broadly into (1) oxidative stress-mediated [27, 28], (2) dysregulated antioxidant mechanisms, (3) inflammation [29, 30], (4) dysregulated lipid metabolism [31–33], and (5) dysregulated angiogenesis [34, 35]. This review focuses on recent developments that explain each of these mechanisms in AMD and in particular describes the various murine models employed in these studies.

## 3. Oxidative Stress and AMD

Oxidative stress appears to be central in the development of AMD due to its relationship with other molecular mechanisms found in AMD. Generally, oxidative stress is characterized by increased levels of reactive oxygen species (ROS) resulting in the damage or modification of cellular proteins, lipids, and DNA, thereby impairing their physiological roles [36]. Several physiologic conditions favor the generation of ROS and oxidative stress in the retina, which includes higher oxygen metabolism, higher polyunsaturated fatty acid content (PFA), and presence of photosensitive molecules (rhodopsin and lipofuscin) and retinal illumination [28].

A huge body of literature supports the involvement of oxidative stress in AMD. Blood serum from AMD subjects showed increased levels of oxidative stress indicated by increased levels of malondialdehyde (MDA), protein carbonyls, and 8-hydroxy-2-deoxyguanosine (8-OHdG) compared to normal non-AMD cohorts [37], thus suggesting that systemic oxidative stress is related to AMD [37]. Concurrently, studies have shown increased oxidative stress in the retina from donors' eyes with AMD [38–41]. Cultured RPE cells from AMD donors' eyes revealed higher ROS production and malfunctioning of the mitochondria [41]. Oxidative damage to mitochondrial and nuclear DNAs was observed in the RPE of AMD subjects [39, 42]. Donor eyes with AMD showed a higher level of the carboxyethylpyrrole (CEP) in the BrM compared to normal eyes [43]. CEP is a lipid peroxidation product formed from docosahexaenoic acid (DHA) under oxidative stress [44]. Since the photoreceptor outer segments are largely composed of DHA [45], the relative increase of CEP in the BrM from donor's eyes with AMD could suggest increased vulnerability to oxidative damage and/or greater exposure to oxidative stress compared to the non-AMD retina. Also, an accumulation of damaged proteins and impairment of autophagy, which is a proteolytic mechanism of efficient antioxidant capability, were observed in AMD [46]. In addition, the finding of cigarette smoking as a major risk factor of AMD in most epidemiological studies emphasized the crucial role of oxidative stress in the development of this retinal disease [47, 48]. The relationship between cigarette smoking and AMD has been demonstrated experimentally in vitro and in wild-type mice and was shown to be directly linked to the oxidants in cigarette smoke [49, 50]. The cigarette smoke extract (CSE) was observed to increase lipid peroxidation by 8-fold in RPE [51]. Investigating the neuroprotection of antioxidants in the development of AMD may be helpful to further entrench the significant role of oxidative stress in AMD as existing literature is inconclusive [52, 53]. Next, we provide a comprehensive review of the animal models to provide insight into the involvement of oxidative stress in the development of AMD.

### 3.1. Cigarette Smoke Model

The exposure of mice to cigarette smoke is an important way to investigate the role of oxidative stress in AMD because cigarette smoking is the most significant modifiable risk factor in AMD [47, 54]. Cigarette smoke contains several potent oxidants, including hydroquinone (HQ), nicotine, and cadmium [55, 56]. This animal model has helped to understand possible molecular events that might lead to AMD. HQ upon entry into the circulation through the lungs diffuses into cells and affects the mitochondria, resulting in the overproduction of superoxide anion and damage to mitochondrial membranes [49]. Leakage of superoxide into the cytoplasm generates ROS, which mediates protein oxidation and lipid peroxidation. In addition, complement activation was found in the cigarette-smoke-mediated retinal degeneration [51, 57].

Espinosa-Heidmann and colleagues demonstrated that HQ from cigarette smoke could cause subretinal deposits, a hallmark of AMD [49]. The protocol for inducement normally involves repeated daily exposure of adult mice to cigarette smoke pumped into sealed chambers for a certain time period of the day. Exposed mice showed elevated serum HQ levels and oxidative stress accompanied by retinal changes including BrM deposits and thickening, inflammation, and choroidal neovascularization [49, 50, 58]. This rodent model shows that oxidative stress could induce other molecular mechanisms to generate the AMD phenotype. The severity of the retinal degeneration was shown to depend on the length of exposure. In a study where mice were exposed to cigarette smoke for 2 hours daily for 4.5 months, there was less damage to the RPE compared to the result from another study which used a 5 hr daily exposure for 6 months [49, 58]. The extended exposure time to cigarette smoke led to apoptosis of the RPE [58].

### 3.2. Light-Induced Model

The damaging effect of light on the retina has been studied and reported to affect mainly the outer retina and RPE [59–61]. Intense retinal illumination has been associated with a reduction in the thickness of the outer nuclear layer and accumulation of deposits within the RPE [62]. Most studies of retinal damage by light have used the BALB/c mice or SD rats, known for their genetic susceptibility to light. Recently, a novel protocol by which retinal degeneration could be induced in the commonly available C57BL/6 J mice was described [63]. The wide use of the light-induced retinal degeneration model for studying AMD is because it offers advantages such as synchronized photoreceptor death occurring with light exposure and is easy to induce within a short time [64]. Also, this model offers the possibility to vary the severity through manipulation of light intensity and duration, and more importantly because light is a natural risk factor involved in many retinal diseases [62].

One of the mechanisms accounting for the retinal damage is due to the interaction between light and photosensitive molecules such as rhodopsin and lipofuscin [59, 65]. The activation of rhodopsin coupled with another cascade of events in the phototransduction process has been associated with photoreceptor cell death [64, 65]. It is likely that such a rhodopsin-mediated mechanism is related to oxidative stress because antioxidant interventions have been found to preserve photoreceptors [66–68]. In addition, light causes the formation of lipid peroxidation from the DHA content of the rods' outer segment membranes [69, 70]. The first light-induced model of retinal degeneration was demonstrated by Noell et al. in albino rats [71]. Since then, several researchers have used similar protocols that differ with respect to the presence of genetic susceptibility of the animal, intensity, wavelength, and duration of light exposure [72–74]. Shorter wavelengths closer to the blue region of the electromagnetic spectrum have been found to have the highest risk for retinal degeneration compared to the longer wavelengths [59]. The susceptibility to retinal damage by the blue light is due to the increased generation of ROS by the photoactivated pigments rhodopsin and lipofuscin in response to this specific range of the electromagnetic spectrum [75, 76].

### 3.3. Carboxyethylpyrrole Immunization Model

**Figure 1:**
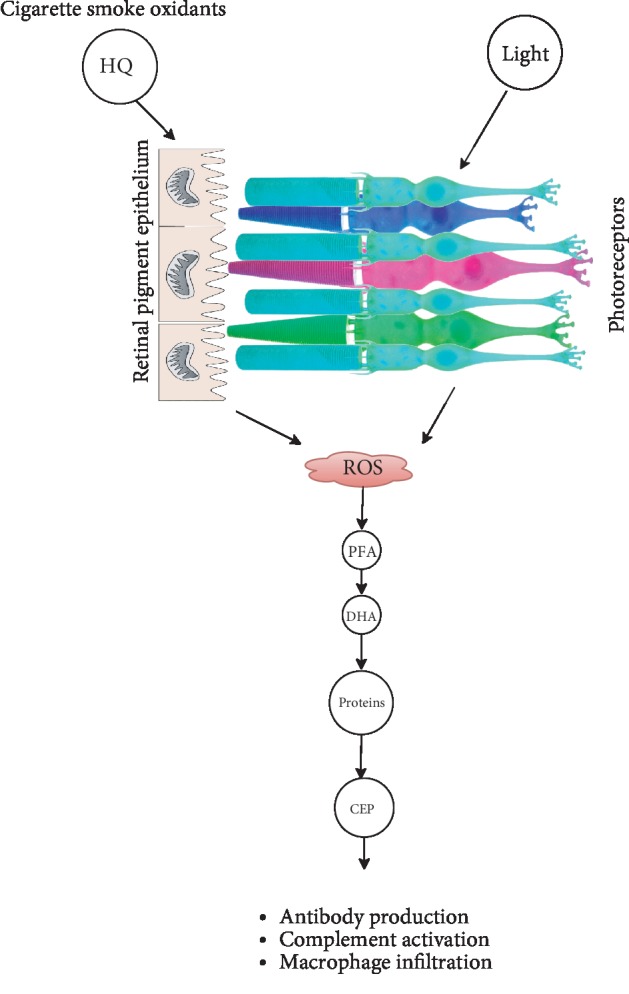
Molecular mechanism in oxidative stress-induced retinal degeneration in mice. ROS levels increase in the retina following (1) exposure to the cigarette smoke prooxidant, hydroquinone (HQ), causing mitochondrial damage in the RPE, and (2) retinal illumination resulting in the photoactivation of rhodopsin. A chain of reactions results in the formation of the carboxyethylpyrrole (CEP) from the docosahexaenoic acid (DHA), a polyunsaturated fatty acid content (PFA) constituent in the retina. CEP is immunogenic, leading to an inflammatory response.

The carboxyethylpyrrole (CEP) model elucidates the mechanism by which oxidative damage to cellular molecules including lipids and proteins could result in inflammation and retinal degeneration. CEP-modified protein adduct is generated by oxidative damage to DHA with subsequent reaction with the lysine moiety of adjacent proteins. CEP protein adducts found in AMD are immunogenic, inducing autoantibody production and inflammation in the retina [43, 77]. The immunization of mice with CEP-modified mouse serum albumin (CEP-MSA) induced antibodies against CEP and led to inflammatory responses such as the deposit of complement component-3 in BrM and macrophage infiltration [78]. Retinal changes observed after 12-14 months of single immunization included loss of RPE, drusen formation, and thickening of the BrM. These retinal degenerative changes could be induced within a shorter duration (i.e., 2-3 months) in mice through repeated immunization. This model may be useful in investigating mechanisms by which oxidative stress may mediate inflammation in AMD. Based on the outcome of the cigarette smoke, light damage, and CEP immunization in mouse's retina, the pathways by which oxidative stress may lead to AMD have been described (Figure 1).

## 4. Dysregulated Antioxidant Mechanisms and AMD

Antioxidants and other antioxidant-related mechanisms play important roles in reducing oxidative stress. Antioxidants are intracellular molecules that scavenge reactive oxygen species (ROS) and enzymes which degrade superoxides and hydroperoxides, protecting against oxidative stress [79]. Vitamins (A, C, and E) and carotenoids (lutein and zeaxanthin) are potent and effective antioxidants essential in retinal function. Carotenoids, in particular, zeaxanthin, are found in the central retina constituting the macular pigment shown to be protective against light-induced oxidative damage through absorption of the near-blue wavelength of light [80]. Vitamin E (*α*-tocopherol) may protect the retina from oxidative damage by acting as a scavenger of lipid peroxyl radicals [81]. Studies have shown that increased antioxidants in diet or serum could be protective against AMD progression. According to a longitudinal clinical study, dietary intake of antioxidant/zinc was found to reduce the risk of early AMD in a highly susceptible population due to genetic polymorphisms of complement factor H (CFH) Y402H and LOC387715 A69S [82]. Although some studies found no protective effect of antioxidants against early AMD [83], there is little controversy over its protective role in late AMD [84]. Also, experimental studies in monkeys have found that the consumption of antioxidant-deficient diet (vitamins A and E, and B carotene deficiencies) was associated with photoreceptor degeneration and lipofuscin accumulation in the RPE [85, 86].

In addition to the antioxidants, autophagy and nuclear factor erythroid 2-related factor 2 (Nrf2) and their associated antioxidant enzymes have been found to be highly beneficial for retinal survival under both normal and adverse conditions [87, 88]. Autophagy is a cellular recycling mechanism possessing efficient antioxidant properties and protective against neurodegenerative diseases [87]. An age-related upregulation of autophagy occurs in the retina of non-AMD donors and mice, indicated by an increase in the number of the autophagosome, autophagy-related proteins, and autophagy flux [46]. In contrast, donor eyes with AMD showed a decline in autophagy, suggesting it may be involved in the disease [46, 89]. One master regulator of the cellular antioxidant mechanism is Nrf2, a transcription factor that regulates the production of antioxidant enzymes against oxidative stress. Under quiescent conditions, Nrf2 is bound to Kelch-like ECH-associated protein 1 (Keap1) in the cytosol, inactive, and predestined for degradation by the ubiquitin-proteasome pathway [90]. However, Nrf2 dissociates from Keap1 under oxidative stress resulting in its upregulation and translocation into the nucleus. This leads to the upregulation of several antioxidant genes and enzymes including heme oxygenase 1 (HO-1), NAD(P)H-quinone oxidoreductase (NQO1), glutathione S-transferase (GST), superoxide dismutase (SOD), ferritin, and glutathione reductase. SOD is a ubiquitous family of enzymes present in all oxygen-metabolizing cells. It constitutes the first line of defense against superoxide radical (O_2_^−^) and other ROS [91]. The O_2_^−^ radical is a highly potent oxidative agent because each free radical rapidly gains three electrons to rebalance itself, unlike other ROS. Also, O_2_^−^ could generate other ROS, particularly hydrogen peroxide and hydroxyl radicals.

In the retina, Cu-Zn (SOD1), Mn-SOD (SOD2), and Fe-SOD (SOD3) are found in the cytosol, mitochondrial matrix, and tissue extracellular space, respectively [92]. Mouse models of retinal degeneration induced by compromising the antioxidant mechanisms provide a platform to understand the role of oxidative stress in AMD.

### 4.1. Autophagy Deficiency Models

The protective role of autophagy against AMD has been demonstrated *in vivo* through the impairment of autophagy to observe any consequential changes in the retina [89, 93]. Yao and colleagues found that the deletion of the RPE gene encoding RB1CC1 (inducible coiled-coil 1) inhibited autophagy in mouse's RPE and accompanied by age-dependent retinal changes including RPE atrophy, microglial infiltration, sub-RPE deposits, and CNV. Subsequently, photoreceptors degenerated and loss of retinal functions occurred [89]. Similar results were observed in mouse's retina following the deletion of RPE-specific Atg5 and Atg7 [93]. Mice aged 8 months old developed retinal signs of early AMD such as abnormal RPE thickness and photoreceptor degeneration [93]. This occurrence of oxidative damage in the retina following the inhibition of autophagy is an indication that autophagy could be an important antioxidant mechanism in the retina. In addition, impairment of autophagy is shown to induce inflammation through the recruitment of inflammasome-activated macrophages [94]. Liu and coworkers showed that impairing autophagy in the eyes of mice via intravitreal injection of wortmannin, an autophagy inhibitor that irreversibly inhibits class III PI3-Kinase12, transiently reduced autophagy activity for one week and led to photoreceptor and RPE death by apoptosis [94]. These findings support the involvement of autophagy in the maintenance of normal homeostasis in the aging retina, and its impairment may play a role in aging retinal degeneration.

### 4.2. Nrf2 Deficiency Model

Retinal degenerative changes, the presence of hard and soft drusen and RPE atrophy, are observed in adult Nrf2 knockout mice by fundus examination [95]. Retinal function assessment revealed a decline in the a-wave and b-wave amplitudes in electroretinograms (ERGs). Microscopic examination of their retinas showed thickening of the BrM and sub-RPE deposits comprising complement components C3d and vitronectin, which are indicators of complement pathway activation. In some eyes, CNV lesions and loss of photoreceptors were also observed. The age-dependent retinal damage occurring in Nrf2-deficient mice elucidates the importance of dysregulated antioxidant mechanisms and oxidative stress in the development of AMD. According to Zhao and coworkers, there was additional downregulation of autophagy in the Nrf2-deficient mice, which they described as an increase in autophagosome and autolysosome and accumulation of oxidatively damaged protein aggregates and organelles [95]. However, the impact of Nrf2 deficiency on autophagy inhibition may require further investigation to be established since increased autophagosome and autolysosome could as well be indications of upregulated autophagy when the stage of binding to these to the lysosomes is uncompromised [96, 97]. Even though this Nrf2 deficiency model may be helpful in addressing some questions in AMD, its use is limited by the fact that the role of Nrf2 in AMD remains unclear. It is yet to be demonstrated whether Nrf2 is differentially expressed in AMD donor eyes compared to normal healthy eyes. However, recent experimental data in mice have also supported the association between Nrf2 deficiency and AMD, as it was found that the Nrf2 mRNA expression under oxidative stress was impaired in the RPE of aged mice compared to younger mice, thus making this association probable in humans [98].

### 4.3. SOD Deficiency Model

The retinal changes occurring in SOD1- and SOD2 knockout mice emphasize a major role of oxidative stress in the pathogenesis of AMD. *Sod1*^−/−^ aged 10 months or older developed retinal drusen, thickened BrM, and CNV. In addition, degenerative changes were found in the RPE and photoreceptors of some mice [99]. Sandbach and colleagues demonstrated that the deficiency in SOD2 expression was associated with an increased mitochondrial ROS production [100]. SOD2 deficiency is lethal, and SOD2 knockout mice die within one week from systemic abnormalities related to oxidative damage [101, 102]. Hence, to study retinal changes arising due to SOD2 deficiency, these mice were treated with the SOD2 mimetic, manganese 5,10,15,20-tetrakis (4-benzoic acid) porphyrin (MnTBAP), which extended their lifespan to 3 weeks. MnTBAP-treated SOD2 knockout mice showed thinning of the inner retinal layer and photoreceptor layer compared to wild type. No change was observed in the RPE and BrM. Perhaps, either the short lifespan of SOD2 knockout mice does not allow sufficient time for additional age-related retinal abnormalities to develop or the MnTBAP treatment for the SOD2 knockout mice have protective properties.

Also, other researchers investigated retinal damage occurring in SOD2 deficiency using a gene therapy approach to overcome the problem of lethality with SOD2 knockout mice [103]. The adeno-associated virus (AAV) expressing a ribozyme gene (Rz432) was administered by subretinal injection into the eyes of adult C57BL/6 mice to target the RPE and knockdown SOD2 expression in adult mice [104]. Eyes treated with Rz432 had reduced SOD2 proteins and increased oxidative damage. At 4 months posttreatment, retinal changes typical of human AMD such as the loss of a- and b-waves amplitudes of ERG, accumulation of oxidized proteins in RPE and degeneration, thickening of BrM, apoptotic photoreceptor death, and increased deposit of A2E, the lipofuscin fluorophore, were observed in most treated eyes [103]. However, the role of SOD in human AMD remains uncertain since an earlier genetic study reporting an association between SOD polymorphism and AMD [105] has recently been challenged [106]. Also, results from a previous study investigating SOD enzyme levels in RPE from donor's eyes with or without macular degeneration found no significant correlations between SOD and aging or macular degeneration [107], making this mechanism only speculative.

### 4.4. *α*-Tocopherol Deficiency Model

*α*-Tocopherol (vitamin E) is a potent fat-soluble antioxidant known for its role as a scavenger of lipid peroxyl radicals. The physiological role of *α*-tocopherol in the body is evident from systemic conditions such as neurological dysfunction, myopathies, and diminished erythrocyte lifespan associated with its deficiency. Following absorption, *α*-tocopherol is transported to parenchymal cells of the liver for storage [108]. The serum concentration of *α*-tocopherol is regulated by *α-*tocopherol transfer protein (*α*-TTP) which is involved in its transport from the liver to other body organs [109]. Mutations in the gene encoding *α*-TTP are linked to ataxia with isolated vitamin E deficiency [109, 110].

Since the retina has a rich lipid content, it was thought that *α*-tocopherol could have an impact. Therefore, to investigate the protective effect of vitamin E on oxidative stress, *α-*TTP knockout mice were generated and fed vitamin E-deficient diet [111]. There was increased lipid peroxidation and degeneration of neurons, and *α-*TTP-deficient mice showed changes in retinal function indicated by attenuation in a- and b-waves in ERG at 12 months, as well as loss of outer and inner segments of photoreceptors by 20 months. This outcome shows that *α*-tocopherol is a potent antioxidant in the retina protective against oxidative stress-related retinal degeneration. However, long-term clinical trials investigating the neuroprotection of vitamin E supplement intake on the development or progression of AMD have consistently found no significant clinical effect [112, 113]. The fact that vitamin E deficiency in humans is rare and often found in isolated cases of abnormal dietary fat absorption or metabolism, instead of a diet low in vitamin E [114, 115], may explain why the intake of it as a supplement may not be of additional benefit in persons with AMD having normal metabolism.

## 5. Inflammation and AMD

Numerous reports have discussed the roles of inflammation in AMD pathogenesis extensively [30, 116]. Analysis of drusen from donated eyes with AMD revealed the presence of complement components C3 and C5 and membrane-attack complex (MAC), suggesting the activation of the complement pathways. In addition, negative regulators of the complement pathways, including vitronectin and clusterin, were also found in drusen [22, 29, 43, 117]. Activation of the complement system causes proinflammatory responses such as the production of MAC, leading to cell lysis and release of chemokines to mediate recruitment of inflammatory cells including microglia and macrophages [29, 118]. The role of inflammation in the pathogenesis of AMD is solidified by genetic studies showing that the Y402H polymorphism of the complement factor H (CFH), a soluble glycoprotein regulating complement activation, is found in more than half of AMD cases and that the presence of this polymorphism is associated with a higher risk of this disease [119–122]. Other indicators of inflammation in AMD are the presence of chemokines and the accumulation of immune cells such as macrophages and microglia in the retina of AMD subjects [123, 124]. Also, the ability to create animal models of retinal degeneration through manipulation of the immune response further highlights the importance of inflammation in AMD. Various animal models supporting inflammation in AMD are discussed in this review.

### 5.1. Chemokine Models

Chemokines and their G-protein-coupled receptors contribute significantly to inflammation in AMD. The role of chemokines in AMD is evident from the increased infiltration of activated macrophages and microglia in the milieu of drusen and atrophic lesions [117, 123]. The enhanced recruitment of these immune cells is due to the differential expressions of chemokines and its receptors in AMD [125, 126]. Chemokines are grouped into four families depending on their conserved cysteine residues: CXC, CX3C, CC, and C [127, 128]. CCL2/CCR2 and CX3CL1/CX3CR1, which are ligand/receptor pairs, have been implicated in macrophages and microglia recruitment, respectively, in AMD [129].

#### 5.1.1. Ccl2^−/−^ or Ccr2^−/−^ Mouse Model

This model presumed that the recruitment of macrophages into the retina was protective. Ambati et al. showed that knocking out *Ccl2/Ccr2*, a chemokine receptor expressed by macrophages and its binding molecule, respectively, in mice led to the inhibition of macrophage recruitment and retinal degeneration. Signs of retinal degeneration found in the adult (16 months and older) mouse retina were drusen, lipofuscin, thickening of the BrM and geographic atrophy, or CNV [130]. These findings, however, have been challenged by others who demonstrated that Ccl2^−/−^ or Ccr2^−/−^ mice showed no change in the thickness of either the BrM or RPE, as well as the photoreceptors [131, 132]. Intriguingly, the same controversy on the involvement of *CCR2/CCL2* in AMD is found in studies using human participants too. A case-control study of the association between *CCL2*/*CCR2* polymorphisms and AMD showed no significant association between these genes and AMD [133]. Furthermore, quantitative PCR reactions evaluating the expression of these genes in laser-dissected RPE from 13 AMD donor eyes and 13 control eyes found no significant difference in the expression of these genes between normal subjects and those with AMD. Controversially, another case-control study with relatively fewer subjects subsequently reported a significant difference in the genotype and allele frequency for *CCL2/CCR2* between AMD and normal controls and concluded that individuals possessing both single nucleotide polymorphisms (SNPs) were at a higher risk of developing AMD [134]. More recently, the demonstration that different functional macrophage subtypes may exist (either protective or injurious) may help to resolve the controversy [135]. Mice given an intravitreal injection of M2 macrophages, a subtype accumulating in wet AMD, displayed exacerbated CNV lesions while mice injected with M1 macrophages displayed ameliorated CNV lesions [135]. The exact role of macrophages and their receptor-ligand pairs (CCR2/CCL2) in AMD require further investigation.

#### 5.1.2. Cx3cr1^−/−^ Mouse Model

Contrary to the proposal that infiltration of macrophages into the retina is protective, the *Cx3cr1^−/−^* mouse model suggests an accumulation of microglia, central nervous system (CNS)-resident macrophages, in the retina is harmful [136]. An in vitro study showed that microglial cells induced the death of photoreceptor cells [137]. Microglia are the first and main form of active immune defense in the CNS [138]. Combadière and colleagues demonstrated that *Cx3cr1^−/−^* mice had impaired microglial egress from the retina, resulting in its accumulation [136]. The *Cx3cr1^−/−^* mice developed sub-RPE deposits, photoreceptor degeneration, and CNV. Recently, a meta-analysis of findings from five long-term studies suggested no association between common *CX3CR1* variants and AMD [139]. Again, no agreement has been reached on the role of CX3CR1 in human population studies.

#### 5.1.3. CCL2^−/−^/CX3CR1^−/−^ Double Knockout Model

The *Ccl2^−/−^/Cx3cr1^−/−^* double knockout murine model was employed to overcome the shortcomings of the longer average time taken for either *Ccl2^−/−^* or *Cx3cr1^−/−^* single knockout mice to express AMD phenotype. The researchers reported success in the creation of a murine model that took between 4 and 6 weeks to exhibit visible drusen-like lesions, as well as histological signs of AMD including thickening of BrM, localized hypopigmentation, and degeneration of RPE, and photoreceptor atrophy [140, 141]. CNV was also observed in some of the mice. In addition, there was an increased deposit of lipofuscin granules and its component N-retinylidene-N-retinylethanolamine (A2E) in the retina of the mice. Also, signs of active inflammation were found in the double knockout mouse model, including complement C3, macrophages, and activated microglia [142]. However, the reproducibility of this murine AMD model is doubtful as works by others have challenged that there could be some other genetic mutation in the breeding pair of mice used [129]. Assuming that the outcomes reported in the different chemokine models were valid, then inflammation may be crucial in the development of CNV and AMD.

### 5.2. Complement Activation Models

Even though several reports pointed to activation of the complement pathways in the AMD retina [22, 117], the discovery that polymorphisms in the gene for CFH were associated with greater susceptibility to AMD demonstrates the contribution of this mechanism to the pathogenesis of this disease [119–122]. One of the reasons for the susceptibility of the Y402H polymorphism of *CHF* in AMD is that the impaired binding of CFH to the BrM results in unregulated complement activation and chronic local inflammation [143]. CFH regulates the complement system by inhibition of the alternative pathway through direct inactivation of C3b or binding to C3b, ultimately inhibiting the synthesis of C3 convertase [144]. Depletion of C3 convertase is necessary otherwise it could lead to MAC formation causing lysis of RPE. CFH reaches the retina mainly by circulation, although some amount is also synthesized by the RPE [145, 146]. Apart from the CFH gene, polymorphisms in *C3* have also been found to be associated with increased susceptibility to AMD [147, 148]. Meanwhile, polymorphisms in complement factor B and complement components 2 (C2) have been found to be protective against AMD [149, 150].

#### 5.2.1. Models of Complement Factor H Deficiency

The role of CFH in inflammation and AMD is supported by the retinal degenerative changes observed in the *Cfh^−/−^* mice and *Cfh*^+/-^ mice. Older surviving *Cfh-/-* mice developed characteristic AMD signs including visual functions and the accumulation of subretinal deposits [151, 152]. Similarly, adult *Cfh+/-* mice fed on a high cholesterol diet developed similar functional and structural changes [152]. These models elucidated the pathophysiological roles of CFH in the retina. The increased sub-RPE deposits in the *Cfh^+/-^* and *Cfh^−/−^* mice were due to the competition between CFH and lipoprotein for binding to the BrM. *Cfh^+/-^* and *Cfh^−/−^* mice, having a deficiency in CFH expression, had lipoprotein accumulation in the BrM forming sub-RPE deposits. They further demonstrated that the *Cfh*^+/-^ mice showed dysregulated activation of complement in the BrM causing inflammation. However, a systemic difference exists between *Cfh^−/−^* mice and *CFH* polymorphism. CFH knockout mice have decreased plasma C3 concentration, unlike *CFH* polymorphisms which do not present with changes in C3 levels [151].

#### 5.2.2. Humanized CFH Mice

This transgenic murine model was developed to evaluate the importance of Y402H polymorphism in AMD. Obviously, a mouse model of AMD based on the Y402H variants, the commonest genetic risk factor found in AMD, would help elucidate the pathological mechanisms of this human disease. To create the Y402H *CFH* transgenic mouse, zygotes from wild-type mice were injected with plasmids containing Y402H variants of human *CFH* to substitute the mouse *Cfh* [153]. Matured transgenic mice aged 12 to 14 months showed drusen-like deposits in the central retina. There was an accumulation of macrophage and microglia, basal laminar deposits, and lipofuscin granules in the retina of these mice. In addition, there were signs of complement activation and inflammation in the retina. The inflammation may be brought about by the reduction in the affinity of CFH to bind to MDA, a lipid peroxidation product arising from oxidative stress in the retina. As such, the free MDA molecules could bind and activate macrophages resulting in inflammation [154]. It is possible that the Y402H polymorphisms lessen the efficiency of CFH to deal with oxidative stress making the aging retina vulnerable to AMD. However, there are conflicting results on the suitability of the humanized *CFH* mice as a model of AMD. Ding et al. generated humanized CFH mice by crossing transgenic mice having a full-length human *CFH* bacterial artificial chromosomes with *Cfh^−/−^* mice [144]. Normal retinal morphology and function were preserved in those humanized *CFH* mice. In fact, even in humans, not all Y402H variants develop AMD. How Y402H polymorphism contributes to AMD remains to be examined.

#### 5.2.3. C3-Overexpressing Mice

The retina from AMD eyes has increased complement expression and activation compared to normal eyes [117]. Therefore, to study whether increased complement activation underlies AMD, adult wild-type mice were administered subretinal injections of murine *C3*-carrying recombinant adenovirus [155]. C3 is a common converging point for the complement pathways, and its breakdown into C3a and C3b initiates the final process leading to the formation of MAC. Scotopic electroretinography showed functional deficits in these exogenous C3-overexpressing mice within 2 weeks. Histology and immunohistochemistry revealed pathological signs including RPE atrophy, loss of photoreceptor outer segments, reactive gliosis, and retinal detachment. The deposition of MAC was observed in the outer segments of photoreceptors. While this model corroborates the role of the complement activation in AMD, its challenge is dealing with the involvement of adenoviruses themselves in the retinal pathological changes. Other than the surgical skill required, it could be a model of choice for investigating therapeutic interventions targeting the complement pathway due to the comparatively shorter duration required for creating this model.

## 6. Dysregulated Lipid Metabolism and AMD

AMD is characterized by the accumulation of sub-RPE deposits including drusen, basal linear, and basal laminar deposits, which are largely composed of lipid [32, 156]. Another evidence supporting dysfunctional lipid metabolism in AMD is based on the association between aging and the accumulation of lipoproteins in the BrM. Lipoprotein accumulation could lead to the formation of a lipid wall, impairing the exchange of nutrients between choriocapillaris and RPE across the BrM and compromising retinal functions [156]. Interestingly, the location of this lipid wall is the same as the sub-RPE deposits found in AMD; possibly, it might be the precursor of these deposits. Lipoproteins found in the retina are either produced locally by the RPE or come from circulation [157]. Lipoproteins transport cholesterol across the BrM to/from the RPE and photoreceptors. Apolipoprotein is the protein constituent found in lipoproteins. Other evidence implicating dysfunctional lipid metabolism in AMD comes from the association between apolipoproteins and AMD [158]. APOE and APOB of low-density lipoproteins (LDL) facilitate lipid metabolism through binding to specific receptors on the liver and other cells. *APOE4* polymorphism is protective against AMD whereas *APOE2* polymorphism is associated with the increased risk of AMD [158, 159]. The protective role of APOE4 has been linked to its increased receptor-binding affinity as compared with APOE2. Thus, APOE4 may facilitate greater lipid metabolism due to its increased binding affinity to the liver, the primary site of lipid metabolism. Others have also reported that APOE4 is associated with higher macular pigment optical density, which also might confer protection against AMD [160]. Also, an association between atherosclerosis and AMD has been reported [161]. Based on the fact that increased serum cholesterol-lipoprotein is a hallmark of atherosclerosis, then by extension, it implicates increased cholesterol in AMD [162]. A direct association between AMD and increased serum cholesterol has been supported by a population-based study showing that higher serum HDL concentration in aged persons doubled their risk of developing AMD [163]. Also, the contribution of dysfunctional lipid metabolism to AMD is upheld by the finding that an intervention modulating lipid metabolism was effective in managing AMD [164]. In this follow-up study of 23 subjects diagnosed with AMD having large soft drusen, a high dose of atorvastatin treatment resulted in a regression of the drusen and vision gain in 10 patients. No subject progressed to advanced neovascular AMD. In addition, the induction of retinal degeneration in animals by the manipulation of genotypes responsible for lipid metabolism further supports the involvement of this mechanism in AMD.

### 6.1. Humanized Apolipoprotein and *Apoe*^−/−^ Mouse Models

Researchers have demonstrated an association between dysfunctional lipid metabolism and AMD in mice through the expression of variant apolipoproteins. It was shown that adult humanized transgenic mice expressing one of the three human APOE isoforms (APOE2, APOE3, OR APOE4) and fed high-cholesterol-containing diets for 8 weeks developed age-related retinal degenerative changes [165]. However, the results sharply contrast the findings from human genome studies which show that APOE4 is protective against AMD [158, 166]. Also, other models such as *Apoe*^−/−^ mice, humanized *APO*^∗^*E3-Leiden* mice, and humanized *APOB100* mice support a relationship between hypercholesterolemia and AMD [33, 167, 168]. All these adult transgenic mice when fed high-fat diets showed hyperlipidemia accompanied by thickening of the BrM and subretinal deposit resembling basal linear deposits which were all age-related except for the Apoe^−/−^ mice [33]. In humans, the APO^∗^E3-Leiden, a defective human APOE-3 variant, was associated with hyperlipoproteinemia (i.e., inability to break down cholesterol and triglycerides) and early onset atherosclerosis [169, 170]. Apolipoprotein B100 (APOB100) is another type of low-density lipoprotein (LDL) involved in cholesterol transport. It is one of the components in sub-RPE deposits in AMD [168]. Several researchers have studied the role of APOB100 in AMD, and most have directly linked the development of retinal degeneration to increased cholesterol accumulation in the retina [171–173]. However, Espinosa-Heidmann and colleagues have contested that there were no obvious increased cholesterol deposits in the BrM and suggested that it is solely due to hyperlipidemia in the APOB100 mice [172]. They showed that the retinal degeneration occurred much faster in younger humanized ApoB100 mice fed a high-fat diet following exposure to blue-green light, suggesting the importance of lipid peroxidation. Furthermore, it was shown that prophylactic treatment of APOB100 mice with subcutaneous injection of the antioxidant tocopherol prevented retinal degeneration in the APOB100 mice. These together reveal that a higher risk of lipid peroxidation in the retina may be a plausible mechanism by which hyperlipidemia may be associated with retinal damage.

### 6.2. *Cd36^−/−^* Mice

The *Cd36^−/−^* mouse model shows the significance of phagocytosis and breakdown of photoreceptor outer segment (POS) by the RPE as a mechanism for maintaining normal retinal homeostasis. CD36, also referred to as fatty acid translocase (FAT), is a membrane glycoprotein used by cells for recognition and binding to specific oxidized low-density lipoproteins, oxidized phospholipids, and long-chain fatty acids, for transport into cells [174, 175]. In the retina, CD36 is abundantly expressed by the RPE and involved in the recognition and phagocytosing of oxidized POS [176, 177]. *Cd36* knockout mice, therefore, have an accumulation of oxidized LDL, thickening of BrM and photoreceptor death [177]. Hence, this model, like the apolipoprotein and *Apoe*^−/−^ mice, supports increased lipid peroxidation in AMD.

### 6.3. *Ldl* Receptor^−/−^ and *Vldl* Receptor^−/−^ Mice

Findings from the *Ldl* receptor^−/−^ and *Vldl* receptor^−/−^ mice enhance our understanding of the association between AMD and dysfunctional lipid metabolism by showing that the lack of LDL/VLDL in mice resulted in changes in the vascular endothelial growth factor (VEGF) expression and retinal neovascularization [178, 179]. The LDL receptors bind to APOB- and APOE-containing lipoproteins to facilitate lipid metabolism [180, 181]. These receptors are abundantly expressed by the liver, to aid in the uptake of cholesterol [182]. Similar to the variant humanized apolipoprotein mice and Apoe^−/−^ mice, mice lacking LDL receptors had increased plasma cholesterol due to impaired cholesterol metabolism [183]. Thickening of the BrM was observed in the *Ldl* receptor^−/−^ mice fed a high-fat diet. In addition, these mice expressed increased levels of VEGF in the outer retinal layers [179]. The VLDL receptors are also involved in the binding and uptake of ApoE-containing lipoproteins. VLDL receptors are expressed in the retinal vascular endothelium and RPE of mice [178]. Interestingly, mice lacking VLDL receptors showed normal serum cholesterol levels but developed retinal degeneration characterized by neovascularization at 2 weeks postnatal, followed by photoreceptor degeneration, RPE hyperplasia, and subretinal fibrosis at the end stage [178, 184]. The results of these mouse models support LDL/VLDL receptors as negative regulators of retinal neovascularization, and interventions targeting them might prove beneficial in the management of wet AMD. The Wnt pathway has been proposed as the mechanism by which VLDL receptors modulate VEGF expression and neovascularization [185].

### 6.4. LDL Injection Model in Rats

This model supports a relationship between hyperlipidemia and AMD by showing that repeated intravenous injections of LDL for 7 days resulted in the accumulation of apolipoprotein B100 in BrM, as well as early AMD-like retinal changes including thickening of BrM, the death of photoreceptors, and inflammation in rat retina [186].

## 7. Dysregulated Angiogenesis and AMD

Angiogenesis describes the formation of new blood vessels from existing blood vessels by either splitting or sprouting [187]. It is essential in development, reproduction, and repair. Dysregulated angiogenesis underlies several human diseases [188]. Progression of AMD may be associated with the development of new choroidal blood vessels into the central retina, an indication of dysregulated angiogenesis [189]. This wet form of AMD characterized by CNV is a major cause of blindness in the elderly. Results from large population-based studies suggest that some populations may be more prone to wet AMD [190]. The formation of new capillaries involves a cascade of events beginning with the degradation of the underlying basement membrane of the existing blood vessel, by the proteolytic activity of the plasminogen activator system and matrix metalloproteinases [191]. This is followed by chemotactic migration and proliferation of endothelial cells into the extracellular matrix stroma, formation of lumen, and maturation of the endothelium [187, 192].

Most evidence implicates VEGF as the proangiogenic factor underlying CNV [34, 35, 193]. These are as follows: (1) vitreous VEGF levels were found to be significantly higher in patients with AMD and CNV compared to healthy controls [194], and (2) clinical trials involving the administration of the anti-VEGF agents, including ranibizumab, bevacizumab, aflibercept, and pegaptanib, markedly suppressed neovascularization and vascular permeability in humans and sustained gain of vision in many AMD patients [195]. However, since VEGF is synthesized *in situ* by the RPE under both normal physiological conditions, it is argued that an equally potent inhibitory regulator must be involved in maintaining homeostasis in the normal retina [196–198]. One antiangiogenic regulator synthesized by RPE is pigment epithelium-derived factor (PEDF). All the regulators are tightly controlled under normal physiological conditions. Conditions such as hypoxia, ischemia, and inflammation promote neovascularization by tilting the balance in favor of increased VEGF expression [199, 200].

In mammals, VEGF binds to 3 VEGF receptors which differ in their affinity and function (VEGFR-1, VEGFR-2, and VEGF-3). The binding of VEGF to VEGFR-2 induces vascular permeability and angiogenesis in vivo [201]. Binding of VEGF to VEGF-R1 may not produce an effect in itself, but VEGFR-1 negatively regulates activation of VEGFR-2 by acting as a decoy receptor that competitively binds to VEGF, thereby modulating the amount of VEGF that binds to VEGFR-2 [202, 203]. VEGFR-3, previously thought to be limited to angiogenesis in the embryo, has been found to be expressed on quiescent vascular endothelial cells in capillaries and is imputed for the induction of human gliomas and colon carcinomas [204, 205]. Further details on the affinity of VEGF receptors to the subtypes of VEGF are comprehensively reviewed by others [206].

### 7.1. Laser-Induced CNV Model

CNV was accidentally discovered as a complication of argon laser photocoagulation treatment of the eye. It was later demonstrated experimentally in monkeys that laser trauma resulting in rupture of the BrM could induce CNV [207]. A CNV-inducement protocol has been described for mice to allow repeatability of outcome [208]. Inflammation occurs following the rupture of BrM by laser trauma which may instigate increased VEGF expression in the retina [209, 210]. Also, Berglin and coworkers demonstrated the involvement of matrix metalloproteinase-2 (MMP-2) in laser-included CNV [211]. They compared the size of CNV lesions created by laser trauma between mice lacking MMP-2 and normal wild-type mice and showed that laser exposure altered the MMP-2 gene and protein expressions resulting in bigger CNV lesions in wild-type mice than the MMP-2 knockout mice. The laser-induced CNV model has helped in the understanding of CNV and led to the development of effective therapy for CNV in human AMD. However, its usefulness is limited due to the substantial damage to the neural retina and BrM by laser treatment.

### 7.2. Light-Induced CNV

Light-induced CNV is another useful model supporting oxidative stress as an upstream mechanism to angiogenesis [212]. Oxidative stress enhances the expression of VEGF and PEDF in RPE, believed to protect against oxidative damage [213, 214]. Albert and colleagues found that repeated exposure of albino rats to intense light for 12 hrs daily for 1 month resulted in an increased level of retinal lipid peroxidation product and retinal changes, including RPE and photoreceptor degeneration, sub-RPE deposits, and CNV [212]. Six months of intense light exposure resulted in complete loss of the outer nuclear layer and the appearance of vast areas of CNV in rats [212]. The progressive retinal changes seen in this light-induced model resemble human AMD and may be appropriate for investigating the pathophysiology of AMD.

### 7.3. VEGF Overexpression Models

These models have been used to investigate the role of VEGF overexpression in CNV through either injection of adenoviral vectors expressing VEGF into the RPE or subretinal injection of microbeads containing RPE, the primary source of VEGF in the retina [215–217]. Spilsbury and coworkers showed that subretinal injection of an adenoviral vector expressing rat VEGF resulted in overexpression of the exogenous VEGF and was accompanied by CNV in the rat eye 2 weeks after injection [216]. At about 3 months there was a loss of RPE and photoreceptors. Similar results were observed by Baffi and coworkers, who used a subretinal injection of an adenoviral vector expressing the human *VEGF* gene in rats [217]. Early retinal changes observed at 4 weeks of postinjection included subretinal exudates and CNV. In addition, there was a shortening of photoreceptor outer segments and reduction of the outer nuclear layer at overlying areas of neovascularization.

Oshima and coworkers, however, argued that increased expression of VEGF alone in vivo could not cause CNV based on the finding that transgenic mice with increased expression of VEGF in RPE had normal retina and choroid [218]. It was further demonstrated that when there was a subretinal injection of a gutless adenoviral vector expressing Ang2, CNV consistently occurred. According to them, VEGF or angiopoietin 2 (Ang2) could not reach the choriocapillaris to induce neovascularization because of the tight junctions between RPE cells. There breaking of the barrier occurs during subretinal injections of the adenoviral vector or microbeads. Also, the adenoviral vectors themselves could induce inflammation [219]. As a result, Wang and colleagues proposed the subretinal injection of an adeno-associated viral vector (AAV) encoding human VEGF in rats [220]. Unlike adenovirus, AAV causes little or no inflammatory response and, hence, not likely to contribute to CNV [221]. While these models provided understanding into the association between increased retinal VEGF and AMD, the compromise of the retinal-blood barrier resulting from the subretinal injection could trigger an inflammatory response and promote the growth of new blood vessels [222].

### 7.4. Matrigel Injection Model

Matrigel is a basement membrane extract that can be used for investigating the roles of different angiogenic substances in CNV [223]. It is liquid at 4°C but solidifies to form a plug following injection into tissues [224]. Matrigel may be composed of structural proteins and growth factors of choice depending on the requirement of an experiment. Implanted Matrigel can also be easily removed from tissue for quantification of angiogenesis by immunohistochemistry or histology. Shen and colleagues demonstrated that the subretinal injection of Matrigel induced CNV and other signs of retinal degeneration in the eyes of mice [225]. Histological examination conducted at week 4 postinjection revealed solid Matrigel located beneath the neuroretina and above BrM. At 12 weeks, RPE and photoreceptor degeneration and various degrees of CNV were also observed. In addition, macrophage infiltration and mild inflammation were seen in some CNV lesions. However, the reported success rate for inducing CNV in mice was low ranging from 30 to 55%.

## 8. Conclusion and Perspective

**Figure 2:**
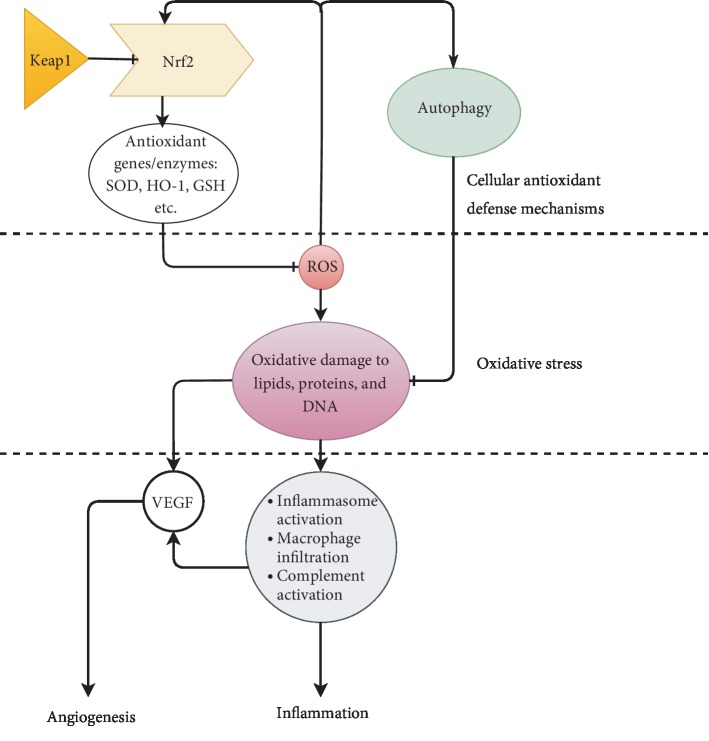
The interrelationships between the molecular mechanisms involved in AMD show the potential therapeutic role of autophagy and Nrf2 activation in the disease. Oxidative damage to lipids, proteins, and DNA is seemingly the primary insult leading to age-related macular degeneration. The accumulation of lipids due to an inhibition of lipid metabolism promotes oxidative stress by increasing lipid peroxidation in the retina. Oxidative stress could initiate inflammation through the activation of the inflammasome, complement, and macrophages. Oxidative stress may also upregulate VEGF expression in the retina and induce choroidal neovascularization. The antioxidant mechanisms, including autophagy and Nrf2, which are upregulated under oxidative stress counteract further oxidative damage and maintain retinal homeostasis.

Rodent models of retinal degeneration provide valuable evidence for the mechanisms involved in AMD and help elucidate how these mechanisms may be interrelated with each other (Figure 2). Oxidative stress appears, however, to be the common link to all the molecular mechanisms. The role of oxidative stress is implicated in the inducement of inflammation in the Y402H polymorphism, which is considered the most important genetic risk of AMD. In the Y402H polymorphism, there is a marked reduction in the ability of the complement regulator CFH to bind to MDA, a lipid peroxidation product [154], leading to uncontrolled MDA-induced inflammation. This is because the binding of MDA to resident macrophages results in the release of cytokines by the macrophages [154], and CFH serves as a decoy to MDA from binding to the macrophages. Thus, the higher risk of AMD associated with Y402H polymorphism is related to an increased vulnerability to oxidative stress in the retina. The role of hyperlipidemia in AMD development also appears to be through increased oxidative stress. Analyzing data from rodent models generated to express the human apolipoproteins shows that these mice showed signs of hyperlipidemia, oxidative stress, and retinal degeneration. The hyperlipidemia (due to impaired lipid metabolism) causes a block of retinal blood vessels and thickening of BrM, leading to ischemia and hypoxia, and the induction of oxidative stress. Last but not the least, photooxidative damage in mouse's eyes has been shown to induce CNV. It is explained that increased oxidative stress promotes increased VEGF production by the RPE cells, as a response to counteract the harm from oxidative stress in the eye. The unregulated VEGF production, however, stimulates the development of new blood vessels. Therefore, targeting VEGF or inflammation (the current focus for AMD treatment), although is proven to be an effective approach, addresses the late stage of AMD. The autophagy and Nrf2 mechanisms are cellular defense mechanisms found to support survival under oxidative stress. The relationship between oxidative stress and autophagy or Nrf2 is bidirectional; increased oxidative stress activates autophagy or Nrf2, and these antioxidant defense mechanisms, in turn, could inhibit oxidative damage in the retina. Since impaired autophagy, Nrf2, or dyslipidemia are factors that could promote oxidative stress in the retina, studies to target these pathways as alternative therapeutic options for AMD are warranted.
